# Sex-Specific Association Between Acute COVID-19 Systemic Inflammation and Persistent White Matter Pathology and Cognition in Survivors

**DOI:** 10.3390/biology15131054

**Published:** 2026-07-02

**Authors:** Mariagrazia Palladini, Mario Gennaro Mazza, Beatrice Bravi, Margherita Bessi, Rebecca De Lorenzo, Patrizia Rovere-Querini, Francesco Benedetti

**Affiliations:** 1 Psychiatry and Clinical Psychobiology Unit, Division of Neuroscience, IRCCS San Raffaele Hospital, 20127 Milano, Italy; mazza.mario@hsr.it (M.G.M.); bravi.beatrice@hsr.it (B.B.); bessi.margherita@hsr.it (M.B.); benedetti.francesco@hsr.it (F.B.); 2 Vita-Salute San Raffaele University, 20100 Milano, Italy; delorenzo.rebecca@hsr.it (R.D.L.); rovere.patrizia@hsr.it (P.R.-Q.)

**Keywords:** COVID-19, inflammation, sex difference, mental health, diffusion-tensor imaging, white matter

## Abstract

Six years into the COVID-19 pandemic, evidence is increasingly clear that long COVID affects women disproportionately, with higher rates of persistent cognitive and neurological symptoms. Yet, the biological mechanisms underlying this sex-dimorphic impact remain elusive. We investigated whether the immune storm of acute COVID-19 leaves a silent yet sex-specific scar on white matter integrity that shapes long-term cognitive health. In 60 previously hospitalized COVID-19 survivors, we combined an inflammatory snapshot at admission proxied by the systemic immune-inflammation index (SII) with 3T diffusion MRI and a comprehensive cognitive battery (BACS) acquired three months after recovery. Sex reshaped the inflammation–brain relationship: a higher SII predicted a diffuse alteration pattern within core associative and inter-hemispheric fibres in females only, sparing the male architecture despite a comparable inflammatory burden. In women, white matter damage coupled with poorer psychomotor coordination, and mean diffusivity fully mediated the link, unveiling a female-specific pathway from systemic inflammation to cognitive slowdown. COVID-19 inflammation imprints a durable, sex-sensitive footprint on white matter that selectively undermines psychomotor coordination in female survivors, despite a clinical recovery. This work positions women’s white matter as a critical target of post-COVID neuroinflammation and argues for sex-informed monitoring and interventions that explicitly tackle immune–brain crosstalk in long COVID.

## Simple Summary

After COVID-19, many people continue to experience fatigue, poor concentration, and memory problems, and these symptoms appear more often in women than in men. However, it is still unclear why this happens biologically. We examined 60 people hospitalized for COVID-19 to investigate whether inflammation during acute infection was linked to later brain changes and cognitive problems. Three months after recovery, we used MRI to examine the brain’s white matter, which is essential for communication between brain regions, and tested thinking, memory, and attention. We found that higher inflammation during acute illness was associated with white matter changes only in women, not in men. In women, these brain changes were linked to a slower performance in tasks requiring speed and coordination. Our results suggest that the immune response during acute COVID-19 can leave a long-lasting, sex-specific effect on the brain, helping explain why women more often report persistent cognitive problems. This may help identify people at higher risk and guide future sex-specific monitoring and treatment for long COVID.

## 1. Introduction

More than five years after the emergence of SARS-CoV-2, the cumulative burden of long COVID is still growing [1]. Under this heading exists COVID-19 survivors who struggle with a puzzling array of symptoms far beyond the virus clearance [2]. As an inherently multi-organ disease, the post-acute sequelae span from cardiovascular, musculoskeletal, immunological, pulmonary, gastrointestinal, and dermatological manifestations to neuropsychiatric issues. Efforts are underway to address the disproportionate impact that long COVID has on women [3]. Consistent evidence pointed out that the female sex increases the vulnerability to residual complications of the illness [4], especially on the neuropsychiatric side [5,6]. Meanwhile, a male bias in mortality for COVID-19 has been documented worldwide.

The immuno-inflammatory hypothesis of long COVID offers a promising framework to address the disparity in the acute and post-acute disease burden. Women are endowed with a more effective cellular and humoral response, as also observed in the prior SARS and Middle East respiratory syndrome [7]. However, the rapid virus clearance in women is achieved at the expense of massive and prolonged autoantibody reactivity, whose extent associates with the presence and timing of post-infection sequelae [8]. A constellation of factors, encompassing sex steroids, sex chromosomes, and sex-specific epigenetic patterns, has been advocated to justify the gender asymmetry in the context of the COVID-19 response to infection and disease progression [9].

While a consensus exists on the inverse epidemiology between COVID-19 severity (worse in males) and post-COVID-19 neuropsychiatric sequelae (worse in females), the biological sex-specific mechanisms upholding the distinct dynamic evolution from the acute viral infection to long COVID issues remain elusive [10,11]. It has been previously demonstrated that measures of both the grey matter (GM) and white matter (WM) volumes and integrity are associated with a long COVID cognitive and depressive psychopathology [12,13,14,15]. A growing body of data suggests that alterations in cerebral perfusion, the brain microstructure, and activation may underpin long COVID cognitive difficulties both subjectively reported and observed regardless of biological sex [16,17]. A more detailed description of the immune–brain interaction underlying neurological consequences in a sex-dependent manner is, however, missing. According to the leading thesis, the women-specific proliferation of autoreactive immune cells in the central nervous system targeting glial and endothelial cells hold the potential to promote a neuroinflammatory milieu [16] and to trigger demyelination/dismyelination, thus driving a detrimental association of persistent low-grade inflammation and the brain microstructure as commonly observed in psychiatric conditions [18,19,20,21], but no direct evidence is available. Experimental immune-challenge paradigms suggest that the affective consequences of inflammation are sex-dependent: women show more depressed mood than men under the same immune challenge, and only in women does the increase in inflammatory markers track with the increase in depressive symptoms [22]. In line with this broader framework, Paolini et al. found that peripheral inflammatory markers were associated with lower hippocampal grey matter volumes in women, but not in men [23].

Together, these findings suggest that the inflammatory burden may be more readily translated into neuropsychiatric and structural brain consequences in women across different clinical contexts, providing a biologically plausible rationale for investigating sex-stratified effects in long COVID. Accordingly, we examined whether acute systemic inflammation is associated with white matter integrity and cognitive performance in COVID-19 survivors at long-term follow-up.

## 2. Materials and Methods

### 2.1. Participants and Measures

We enrolled 60 participants (44 males, age 54.17 ± 9.75) from San Raffaele Hospital (Milan, Italy). The sample has been collected starting from March 2020 until December 2022 in the context of an ongoing prospective cohort study taking place at San Raffaele Hospital. They all have been hospitalized for COVID-19, and then they have been tested over three months after full recovery. Clinical and socio-demographic information was assessed through an unstructured clinical interview conducted by well-trained psychologists. Inclusion criteria were being over 18 years old and being diagnosed with COVID-19 infection, as suggested by radiological findings at the emergency department (ED) and further corroborated by positive real-time reverse-transcriptase polymerase chain reaction (RT-PCR) from a nasopharyngeal and/or throat swab. Excluding criteria were as follows: intellectual disabilities, history of drug or alcohol use disorder within the last six months, major neurological disorders, and pregnancy. When admitted to emergency department they underwent a blood sample, thus inflammatory markers of acute COVID-19 infection were derived. We computed systemic inflammation index (SII), an objective marker of the balance between host systemic inflammation and immune response status, considering the neutrophil, platelet, and lymphocyte together (SII = platelets X neutrophils/lymphocytes). At the follow-up, the sample underwent MRI, and their cognitive functions were assessed through test Brief Assessment for Schizophrenia (BACS), a battery testing a variety of cognitive functions: verbal memory, verbal fluency, selective attention, processing speed, working memory, psychomotor coordination, and executive functions. This test provided us a scoring on each specific cognitive domain and, at the mean time, a score on global cognitive functioning through the mean of each score. Domain raw scores were adjusted for age, sex, and education according to normative Italian scores for the BACS subtests [24]. The follow-up coincided with MRI scan test, through which we conducted Diffusion Tensor Imaging (DTI) analysis. After a complete description of the protocol, written informed consent was obtained.

### 2.2. DTI Data Processing and Statistical Methods

All imaging was performed on a 3.0 T scanner (Ingenia CX, Philips, The Netherlands) with spin-echo echo-planar imaging (EPI) and the following parameters: TR/TE = 5900/78 ms, FoV (mm) 240 (ap), 129 (fh), 232 (rl); acquisition matrix 2.14 × 2.73 × 2.30; 56 contiguous, 2.3 mm thick axial slices reconstructed with in-plane pixel size 1.88 × 1.88 × 2.30 mm; SENSE acceleration factor = 2; 1 b0 and 40 non-collinear directions of the diffusion gradients; and b value = 1000 s/mm2. Fat saturation was performed to avoid chemical shift artifacts. Image analyses and tensor calculations were done using the “Oxford Center for Functional Magnetic Resonance Imaging of the Brain Software Library” (FSL 6.0; www.fmrib.ox.ac.uk/fsl/index.html, accessed on 16 January 2026) [25]. Each DTI volume was affine-registered to the T2-weighted b = 0 volume using FLIRT (FMRIB’s Linear Image Registration Tool) [26]. Correction for susceptibility-induced off-resonance field, eddy-current-induced distortions, and subject movements was performed [27]. Before preprocessing, raw DWI data were visually inspected for gross artifacts. In addition, EDDY provides diagnostic outputs and detects slice/volume outliers, which can be modelled and replaced within the reconstruction when appropriate. Least-square fits were performed to estimate the fractional anisotropy (FA), eigenvector, and eigenvalue maps. Mean diffusivity (MD) was defined as the mean of all three eigenvalues (λ 1 + λ 2 + λ 3)/3, axial diffusivity (AD) as the principal diffusion eigenvalue (λ 1), and radial diffusivity (RD) as the mean of the second and third eigenvalues (λ 2 + λ 3)/2. Next, all individuals’ volumes were skeletonized and transformed into a common space as used in Tract-Based Spatial Statistics [28]. Briefly, all volumes were nonlinearly warped to the FMRIB58_FA template supplied with FSL (http://www.fmrib.ox.ac.uk/fsl/tbss/FMRIB58_FA.html, accessed on 16 January 2026) and normalized to the Montreal Neurological Institute (MNI) space. Next, a mean FA volume of all subjects was generated and thinned to create a mean FA skeleton representing the centres of all common tracts. Individual FA values were warped onto this mean skeleton mask. The resulting tract invariant skeletons for each participant were fed into voxel-wise permutation-based cross-subject statistics. Similar warping and analyses were used on MD, AD, and RD data. Voxel-wise DTI analyses were performed using nonparametric permutation-based testing as implemented in Randomise in FSL. Within a GLM framework, we modelled an interaction between sex and SII values at emergency department to explore whether the burden of systemic inflammation during the acute phase of infection differently affect WM structure in men and women in the long run. To account for baseline differences between groups, all TBSS analyses were adjusted for age, BMI, clinically relevant depressive symptoms, and length of hospital stay. Post hoc analyses in each group were then run to investigate how SII values impact fibres’ integrity. As a sensitivity analysis, the TBSS models were repeated in a subsample matched for age and frequency of depressive syndrome (women, *n* = 16; men, *n* = 22).

Threshold-free cluster enhancement (TFCE) was used to avoid defining arbitrary cluster forming thresholds and smoothing levels [29]. Voxel-wise levels of significance, corrected for multiple comparisons, were then calculated with standard permutation testing by building up the null distribution (across permutation of the input data) of the maximum (across voxels) TFCE scores, and then using the 95th percentile of the null distribution to threshold signals at corrected *p* < 0.05. The data were tested against an empirical null distribution generated by 5000 permutations for each contrast, thus providing statistical maps fully corrected for multiple comparisons across space. Corrected *p* < 0.05 in a minimum cluster size of k = 100 was deemed significant.

Secondly, we explore the potential interacting effect of DTI measures with sex in predicting cognitive performance in BACS domains, only for WM parameters that were deemed significant in whole-brain analysis. With this aim, we extracted DTI values from significant WM tracts resulting from TBSS model. Adjusted BACS domain scores, based on Italian normative conversion tables accounting for age, sex, and education, were entered as dependent variables in a multivariate GLM model (MANCOVA), with the DTI × sex interaction term as the effect of interest and with depressive symptomatology, length of hospitalization, and BMI included as nuisance covariates. For BACS subtest reaching significance at univariate level, post hoc GLM model was run to disentangle the association with DTI measures in the two groups separately. The effect of predictors was modelled in the context of General Linear Model, and statistical significance of the effect was computed by parametric estimates of predictor variables (least-squares method). All the statistical analyses were performed with a commercially available software package (StatSoft Statistica 12, Tulsa, OK, USA).

Lastly, we tested whether WM measures mediated the association between systemic inflammation and cognitive issues. Mediation analyses were performed only in the subgroup for which the prerequisite associations were present, namely, a significant association between the independent variable and the mediator, and a significant association between the mediator and the outcome. Accordingly, the mediation model was tested for women only using PROCESS macro for IBM SPSS Statistics, version 4.0 (Hayes, 2021; IBM Corp., Armonk, NY, USA; https://www.processmacro.org/index.html; accessed on 9 March 2026). We obtained 95% confidence percentile intervals of effects via 5000 bootstraps. The R^2^ was calculated as mediation effect size measure, while the completely standardized indirect effect was used to evaluate the effect size of the indirect effect.

## 3. Results

The clinical and demographic characteristics of the sample are reported in Table 1. The females were younger than men; yet, the two groups were comparable for most socio-demographics. The length of hospital stay was marginally longer in men, who generally develop a more severe COVID-19 illness. According to the literature, higher frequencies of clinical depression were observed in females [30]. Sex groups reported similar BACS scores on almost any subtest, with a marginal trend toward poorer psychomotor coordination in females (Table 1).

**Figure 1:**
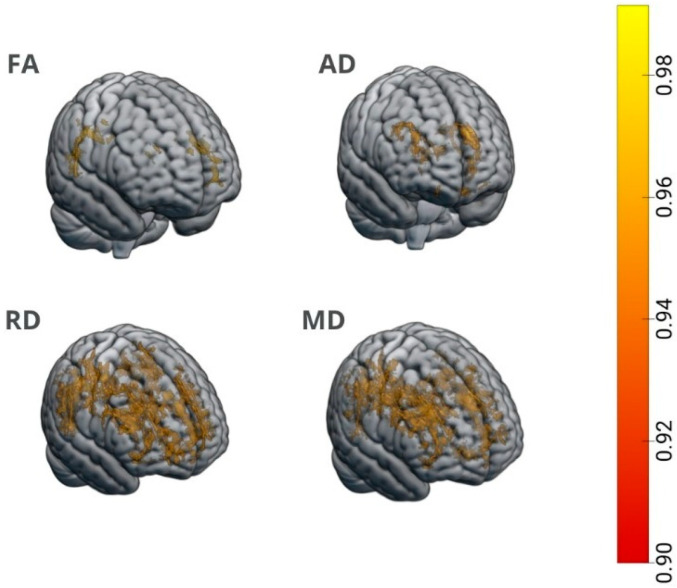
White matter (WM) tracts where systemic inflammation (SII levels) negatively impacted fractional anisotropy (FA, **top left**), while positively associating with axial diffusivity (AD, **top right**), radial diffusivity (RD, **bottom left**), and mean diffusivity (MD, **bottom right**) measures in the white matter skeleton of females. The color bar shows *p* (1 − *p*) values for the association at TFCE analysis.

The TBSS results showed that sex significantly influenced the association between SII and DTI measures, resulting in a marked interactive effect on the axial and mean diffusivity in a large portion of the WM skeleton, mainly entailing the corpus callosum and cortico-thalamic projecting fibres (Table A1). Post hoc analyses showed a detrimental impact of acute systemic inflammation on WM integrity in women, as proxied by a reduction in FA together with an increase in AD, RD, and MD in the widespread portion of the WM skeleton proportional to the inflation of acute SII values (Table A2). Conversely, no association of systemic inflammation on DTI measures was observed in men (Figure 1). Sensitivity analyses in the age- and depressive-symptom-matched subsample (women, *n* = 16; men, *n* = 22) fully confirmed the sex*SII pattern across all white matter measures, with no significant post hoc association in men (Table A3 and Table A4).

**Figure 2:**
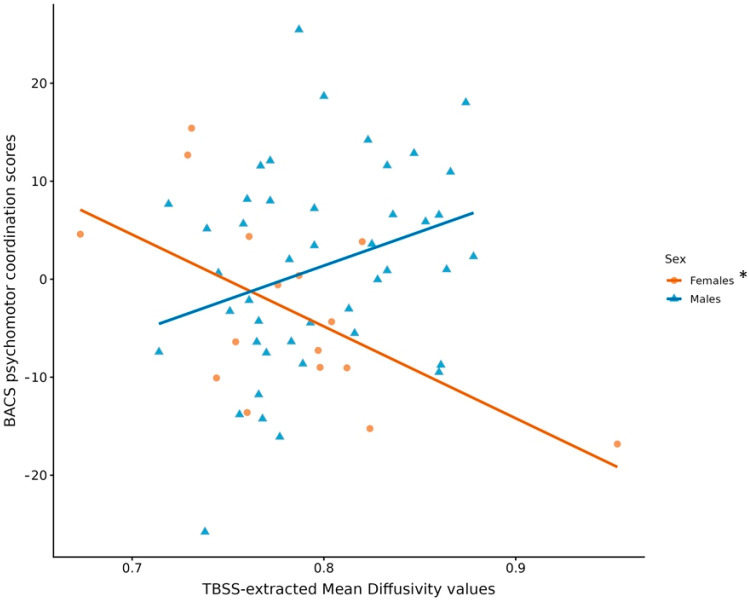
Sex-specific association between TBSS-extracted mean diffusivity (MD) and BACS psychomotor coordination scores. The association was significant in females and showed a trend in males. * Denotes the significant post hoc effect in females.

Furthermore, the overall interaction between MD values and sex on BACS scores was not significant in the multivariate model. However, at the univariate level, a strong sex-modulating role in affecting the association between systemic inflammation and psychomotor coordination scores emerged (F = 9.17, *p* = 0.004, pFDR = 0.032). Post hoc analyses revealed that, in women only, higher MD values were associated with poorer psychomotor coordination performance (β = −0.59, F = 11.45, *p* = 0.006, pFDR = 0.032), while an opposite trend was detected for men (β = 0.35, F = 5.79, *p* = 0.02, pFDR = 0.054) (Figure 2).

**Figure 3:**
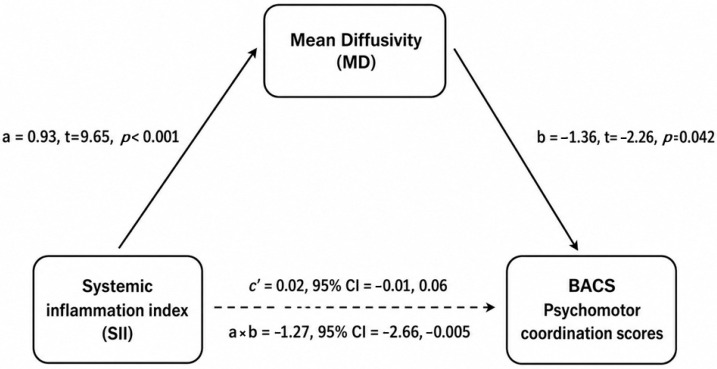
Mediation model of the effect of SII on psychomotor coordination, mediated by mean diffusivity of white matter in females. a, b, a×b: coefficients of the effect of each factor (a and b) and their combined effect (a×b) on outcome, with T statistic or confidence intervals as appropriate.

Based on these findings, mediation analysis was conducted in women only to test whether the relationship between the SII and psychomotor coordination score was mediated by MD values, correcting for the same set on nuisance covariates as in previous analyses. The analyses confirmed the substantial impact of acute systemic inflammation on the MD parameter, highlighting a significant effect of the mediator on the psychomotor coordination scores accounting for the baseline SII as well. The model revealed the significant indirect effect of SII on psychomotor coordination mediated by MD (a×b = −1.27, 95% CI = −2.66, −0.005), whilst the direct effect was not (c’ = 0.02, 95% CI = −0.01, 0.06), suggesting a full mediation model (Figure 3).

## 4. Discussion

This exploratory study addresses the pathophysiology of long COVID neurocognitive issues, by integrating measures of systemic inflammation, WM integrity, and cognitive performances with a sex-disaggregated approach. Our findings suggest that, in females but not in males, markers of acute systemic inflammation during COVID pneumonia still negatively impact cognitive functioning by acting on the WM microstructure months after the resolution of the acute COVID-19 illness. Inflammation-related WM disruption, as proxied by the widespread increased mean diffusivity, was associated with lower scores in psychomotor coordination. This effect could contribute to the explanation of why previous reports showed significant interactions of sex with psychopathology in influencing COVID-19’s detrimental effects on cognition [31].

The higher vulnerability of the human female brain to the detrimental effects of inflammation on brain structure, mood, and cognition is not unique to post-COVID conditions. Females are more affected than males by the detrimental effect of inflammation on mood, long-term fatigue, and chronic pain, even when the increase in inflammatory biomarkers is similar [32,33]. Brain imaging studies assessing sex differences in the human brain response to inflammation are few, but our observation is in agreement with the previous sparse findings in psychiatric and neurological conditions, showing worse hippocampal volumes, and worse depression, as a function of peripheral inflammation in females, but not in males [23]; higher CRP levels are more strongly associated with lower GM volumes and lower FA in healthy aging females than in males [34], and different profiles in cortical cytokines and inflammasome proteins in females and males during aging in animal models [35]; higher cytokines, and higher sensitivity to inflammatory and CBF changes, in female patients with acute-stage mild traumatic brain injury [36]; a worse reduction in grey and white matter volumes in female patients with Systemic Lupus Erythematosus [37]; more gadolinium-enhancing lesions, indicative of a more brain inflammatory phenotype, in females with multiple sclerosis than in males [38]; and a higher sensitivity to the cumulative effect of stress exposure on the WM microstructure in healthy females than in males [39].

Several mechanisms could underlie this apparent sex-specific vulnerability. Studies of the WM microstructure in adult healthy humans usually show a higher FA in males than in females, suggesting a more tightly packed WM and greater WM microstructural coherence, an effect driven by sex hormone exposure and not by karyotype [40]. The major lifetime changes in estrogen availability result in changes in the WM microstructure in females, an effect associated with cognitive function [41]. It can be surmised that this higher WM plasticity in females could also confer a specific vulnerability to insults. These mechanisms are paralleled by a different reactivity to inflammatory triggers. The experimental induction of low-grade inflammation triggers a higher immunoreactivity and worse behavioral consequences in vivo in healthy human females [33]. Once more, the vulnerability to the peripheral and central effects of the experimental induction of inflammation seems to be influenced by hormonal availability, with major lifetime changes in females associated with a history of pregnancies, or hormonal administration [32]. The understanding of sex-specific neuroinflammation has been limited by the paucity of sex-stratified clinical analyses and by the underrepresentation of females in preclinical models, but the available data suggest that sex-based differences in immune system lifetime dynamics and CNS surveillance contribute to divergent trajectories of neuroinflammation: the stronger innate and adaptive immune responses in the aging female can lead to worse brain damage [42]. In addition to the possible specific effects of pathogens, heightened immune-inflammatory responses in females have been also observed in response to psychosocial stressors [32].

In the specific case of the response to COVID-19, it should be noted that the presence of the Angiotensin Converting Enzyme 2 (ACE-2) encoding gene on the X chromosome results in the overexpression of the protein in women [43], capable of counteracting the virus-induced ACE-2 downregulation and ensuing pro-inflammatory and pro-fibrotic activity of the renin angiotensin system (RAS) [44]. Likewise, estrogens in the female gender additionally act as facilitators for ACE-2 synthesis and serve as boosters for immune reactivity also in the context of COVID-19 [43]. Furthermore, the pivotal role of sex steroids in shaping the vulnerability to long COVID sequelae, especially towards neurocognitive issues, is further strengthened by the absence of such a gap in early childhood and late adulthood, with the greatest divergence in the burden of cognitive dysfunction among survivors of reproductive age [3,45].

These mechanisms could all contribute to worse brain structural and cognitive effects in females observed in the present study and stress the need to address hormones and women’s health to understand the pathophysiology of post-COVID depression [3]. Our results should be viewed in light of some limitations. The monocentric recruitment in a single ethnic group could affect the possibility of population stratifications limiting the generalizability of the findings. In addition, the available sample size was insufficient to model potential higher-order interactions among biological, clinical, and pharmacological variables, including the comorbidities and heterogeneity of acute-phase treatments, which may have influenced the SII and MRI measures. Although sensitivity analyses in an age- and depressive-symptom-matched subsample supported the robustness of the main findings, larger studies with more balanced sex and clinical distributions will be needed to fully exclude residual confounding. Finally, a word of caution is needed because of the correlational nature of neuroimaging studies, thereby hampering the interpretation of the neurobiological basis of WM MRI measures. Future studies should compare COVID-19 survivors with appropriately matched non-COVID controls assessed with the same inflammatory, neuroimaging, and cognitive measures, to clarify the extent to which these associations are specific to COVID-19-related inflammation.

## 5. Conclusions

Our findings suggest a plausible sex-specific biological pathway linking acute COVID-19 inflammation to persistent white matter injury and slowed psychomotor performance in female survivors. By showing that the post-COVID brain burden may be not only durable but also sex-dependent, this study supports the sex-informed monitoring and mechanistic research into long COVID.

## Figures and Tables

**Table 1:** Table reports demographic and clinical data of patients, each specific cognitive domain tested through BACS, and systemic inflammation index collected through blood charts during hospitalization (infection’s acute phase). Each variable was investigated, stratifying for sex (via *t*-test or chi-squared as appropriate), and all significances are reported (*p* < 0.05 are specified with *).

Variables	Whole Sample	Men (*n* = 44)	Women (*n* = 16)	t or χ^2^	*p*-Value
Age	54.17 ± 9.75	56.14 ± 9.82	48.75 ± 7.46	2.73	0.008 *
Body mass index	26.98 ± 3.99	27.15 ± 2.83	26.52 ± 6.29	0.53	0.598
Time elapses between infection and MRI scan	138.52 ± 150.88	144.93 ± 159.25	120.88 ± 128.04	0.54	0.589
Length of hospital stay	17.4 ± 16.57	20.43 ± 18.03	9.06 ± 7.8	2.43	0.018 *
Clinically relevant depressive symptoms—Yes (%)	17/60 (18.28%)	8/44 (18.19%)	9/16 (56.25%)	8.37	0.004 *
Adjusted verbal memory scores	46.37 ± 9.63	46.44 ± 9.47	46.19 ± 10.5	0.09	0.93
Adjusted verbal fluency scores	36.58 ± 9.43	37.28 ± 10.09	34.67 ± 7.21	−0.95	0.347
Adjusted speed of information processing scores	51.85 ± 9.08	51.68 ± 9.24	52.33 ± 8.9	−0.24	0.809
Adjusted working memory scores	19.41 ± 4.34	17.96 ± 4.35	17.91 ± 4.06	1.64	0.107
Adjusted executive functions scores	15.81 ± 3.85	16.06 ± 4.24	15.12 ± 2.45	0.84	0.419
Adjusted psychomotor coordination scores	77.35 ± 10.76	78.87 ± 10.55	73.18 ± 10.55	1.847	0.07
BACS mean scores	2.07 ± 0.65	2.11 ± 0.64	1.96 ± 0.68	0.82	0.418
Systemic inflammation index (SII)	1803.84 ± 1873.49	1802.96 ± 1362.75	1806.3 ± 2912.46	−0.01	0.995

## Data Availability

The data that support the findings of this study are not openly available due to reasons of sensitivity and are available from the corresponding author upon reasonable request.

## Appendix A

**Table A1:** WM tracts where significant interaction occurs between baseline systemic inflammation index and sex on AD and MD.

DTI Measures	Cluster Peak Coordinates	Number of Voxels	Peak	WM Tracts Included in the Cluster
**Axial diffusivity** **(AD)**	27 −17 27	1466	Superior corona radiata (right)	Body and splenium of corpus callosum Anterior and posterior limb of internal capsule (right) Retrolenticular part of internal capsule (right) Superior and posterior corona radiata (right) Posterior thalamic radiation (right) External capsule (right) Superior longitudinal fasciculus (right)
	−12 −17 11	760	Anterior thalamic radiation (left)	Fornix
	−20 19 27	639	Anterior corona radiata (left)	Body of corpus callosum Anterior and posterior corona radiata (Left)
	−25 −32 28	219	Posterior corona radiata (left)	Splenium of corpus callosum Retrolenticular part of internal capsule (left) Superior and posterior corona radiata (left)
**Mean diffusivity** **(MD)**	−4 14 20	471	Body of corpus callosum	Genu and body of corpus callosum Anterior and superior corona radiata (right)
	26 21 28	138	Anterior corona radiata (right)	Anterior corona radiata (right)

**Table A2:** WM tracts where baseline SII values significantly affect WM measures in the group of women. A detrimental impact of SII on FA values was found, whereas all the others DTI parameters are positively associated with SII.

	Cluster Peak Coordinates	Number of Voxels	Peak	WM Tracts Included in the Cluster
**Fractional anisotropy** **(FA)**	22 −56 30	1466	Inferior fronto-occipital fasciculus (right)	Inferior fronto-occipital fasciculus (right) Splenium of corpus callosum Posterior corona radiata (right) Posterior thalamic radiation (right) Cingulate gyrus (right) Superior longitudinal fasciculus (right)
	−35 16 18	677	Inferior fronto-occipital fasciculus (left)	Inferior fronto-occipital fasciculus (left) Superior longitudinal fasciculus (left)
	−40 −6 28	244	Superior longitudinal fasciculus (left)	Superior longitudinal fasciculus (left)
**Axial diffusivity** **(AD)**	−25 15 29	1174	Anterior corona radiata (left)	Body of corpus callosum Anterior limb of internal capsule (left) Anterior corona radiata (left) Superior corona radiata (left)
	23 −8 34	917	Superior corona radiata (right)	Anterior corona radiata (right) Superior corona radiata (right) Posterior corona radiata (right)
	−11 −18 13	451	Anterior thalamic radiation (left)	Anterior thalamic radiation (bilateral) Fornix (stria terminalis)
	−26 −34 26	422	Posterior corona radiata (left)	Splenium of corpus callosum Retrolenticular part of internal capsule (left) Superior corona radiata (left) Posterior corona radiata (left) Posterior thalamic radiation (left) Superior longitudinal fasciculus (left)
	11 −20 9	348	Anterior thalamic radiation (right)	Anterior thalamic radiation (right) Fornix (body)
	−23 22 5	241	Anterior corona radiata (left)	Anterior limb of internal capsule (left) Anterior corona radiata (left) External capsule (left)
**Radial diffusivity (RD)**	−15 20 −21	19,741	Genu of corpus callosum	Genu, body, and splenium of corpus callosum Anterior corona radiata (bilateral) Superior corona radiata (bilateral) Posterior corona radiata (bilateral) Posterior thalamic radiation (right) Inferior longitudinal gyrus (right) External capsule (bilateral) Cingulate gyrus (right) Superior longitudinal fasciculus (bilateral)
	15 −16 12	1311	Anterior thalamic radiation (right)	Anterior thalamic radiation (right) Fornix (bilateral)
	−24 −58 43	1214	Superior longitudinal fasciculus (left)	Posterior corona radiata (left) Superior longitudinal fasciculus (left)
**Mean Diffusivity** **(MD)**	−17 19 −19	16,449	Splenium of corpus callosum	Genu, body, and splenium of corpus callosum Anterior limb of internal capsule (left) Posterior limb of internal capsule (right) Retrolenticular part of internal capsule (bilateral) Anterior, posterior, superior corona radiata (bilateral) Posterior thalamic radiation (bilateral) External capsule (bilateral) Superior longitudinal fasciculus Superior fronto-occipital fasciculus (left)
	15 −16 12	1489	Anterior thalamic radiation (right)	Anterior thalamic radiation (right) Fornix (bilateral)

**Table A3:** Table reports demographic and clinical data of patients, and systemic inflammation index collected through blood charts during hospitalization (infection’s acute phase) for the age- and depressive-syndrome-matched subsample. Each variable was investigated, stratifying for sex (via *t*-test or chi-squared as appropriate).

Variables	Whole Sample	Men (*n* = 22)	Women (*n* = 16)	t or χ^2^	*p*
Age	49.45 ± 8.67	49.95 ± 9.61	48.75 ± 7.43	0.42	0.678
Body Mass Index	27.29 ± 4.79	27.84 ± 3.37	26.52 ± 6.29	0.84	0.408
Time elapses between infection and MRI scan	121.08 ± 118.16	121.23 ± 113.53	120.88 ± 128.04	0.009	0.993
Length of hospital stay	14.42 ± 15.15	18.31 ± 17.98	9.06 ± 7.8	1.93	0.062
Clinically relevant depressive symptoms—Yes (%)	17/38 (39.53%)	9/16 (56.25%)	8/22 (36.37%)	1.48	0.224
Systemic inflammation index (SII)	1556.42 ± 2035.22	1374.69 ± 1075.63	1806.3 ± 2912.64	−0.64	0.526

**Table A4:** Summary of TBSS results obtained in the age- and depressive- syndrome-matched subsample. TFCE-derived p-values are reported for both interaction (Sex*SII) and post- hoc models across all WM metrics. Up-ward/downward arrows indicate positive and negative association between SII and WM metrics respectively.

DTI Measures	TFCE-Derived *p* Values for Sex*SII	TFCE-Derived *p* Values for Males’ Post-Hoc Regression on SII
**Fractional anisotropy** **(FA)**	0.017	↑ 0.403 ↓ 0.285
**Axial diffusivity** **(AD)**	0.007	↑ 0.603 ↓ 0.08
**Radial diffusivity (RD)**	0.009	↑ 0.367 ↓ 0.435
**Mean Diffusivity (MD)**	0.01	↑ 0.4936 ↓ 0.284
